# Optimizing tendon repair and regeneration: how does the *in vivo* environment shape outcomes following rupture of a tendon such as the Achilles tendon?

**DOI:** 10.3389/fbioe.2024.1357871

**Published:** 2024-02-16

**Authors:** David A. Hart, Aisha S. Ahmed, Junyu Chen, Paul W. Ackermann

**Affiliations:** ^1^ Department of Surgery, Faculty of Kinesiology, McCaig Institute for Bone and Joint Health, University of Calgary, Calgary, AB, Canada; ^2^ Department of Molecular Medicine and Surgery, Karolinska Institutet, Stockholm, Sweden; ^3^ Department of Orthopedics, Orthopedic Research Institute, West China Hospital of Sichuan University, Chengdu, China

**Keywords:** tendon repair, tendon regeneration, *in vivo* environment, immobilization, induction of atrophy, inflammation

## Abstract

Risk for rupture of the Achilles tendon, and other tendons increases with age. Such injuries of tissues that function in high load environments generally are believed to heal with variable outcome. However, in many cases, the healing does not lead to a good outcome and the patient cannot return to the previous level of participation in active living activities, including sports. In the past few years, using proteomic approaches and other biological techniques, reports have appeared that identify biomarkers that are prognostic of good outcomes from healing, and others that are destined for poor outcomes using validated criteria at 1-year post injury. This review will discuss some of these recent findings and their potential implications for improving outcomes following connective tissue injuries, as well as implications for how clinical research and clinical trials may be conducted in the future where the goal is to assess the impact of specific interventions on the healing process, as well as focusing the emphasis on regeneration and not just repair.

## Introduction

Tendons are complex tissues, consisting of a myotendinous junction, a mid-substance, and an insertion into bone. They are also heterogenous, existing in a variety of environments with differing mechanical requirements, differing fine structures, functioning in collaboration with a sheath or not, and changing with age. Unlike many ligaments which function in more in the toe region of the stress-strain curve, many tendons function in high load environments.

Functioning in high load environments increases risks for developing chronic conditions such as tendinopathies with accompanying pain and loss of function. The high load environment lead to a great metabolic demand, which results in that tendons are vulnerable to slight metabolic disorders (Ackermann and Hart, 2016). As humans age, many tendons become stiffer [reviewed in Kwan et al. (2023)], and can lead to increased risk for tendon ruptures, such as for the Achilles tendon (AT) which functions in a high load environment and is an energy-returning tendon. Tendons, particularly the flexor tendons of the hand are frequently damaged or severed due to trauma. In both situations, the tissue often requires surgery to reconnect the torn ends in order to facilitate repair [discussed in Svedman et al. (2018)].

The outcomes of such repair surgery can be varied, in part, depending on the location and environment, but also on other factors such as genetics, epigenetics, co-morbidities (i.e., diabetes), age, and expectations of future use, such as a return to sport participation. Thus, repair of a tendon such as a flexor tendon of the hand that functions in the context of a sheath, adhesions can develop post-surgery leading to loss of function [discussed in Kuroiwa and Amadio (2023)], a condition that can be influenced by a variety of interventions (Wiig et al., 2014; Edsfeldt et al., 2017; Jiang K. et al., 2023). In the case of the AT, some individuals heal naturally after surgery with a long-term good outcome, while others provided the same surgical procedure ± later *versus* early loading experience a much less satisfactory outcome [(Addevico et al., 2019; Chen et al., 2021; Saarensilta et al., 2023a); discussed in (Hart et al., 2023)]. However, even with surgery the repaired tendon may still be compromised at 2 years post-surgery (Geremia et al., 2015), and thus functional repair likely does not yield regeneration. Furthermore, the tendon-muscle unit may not return to normal even after 10 years (Lantto et al., 2015).

While some aspects of outcomes may be related to “good genes”, in addition the local environment after the initial injury could be contributing to long-term outcomes. The local injury likely induces an inflammatory response, and certainly a follow-up surgery to repair the tissue would also be pro-inflammatory, and inflammation would need to be regulated carefully to allow for successful healing. This of course would be acute inflammation, and if it was prolonged and became chronic inflammation, there could be adverse consequences to outcomes. Relevant to this point are previous studies where glucocorticoid (GC) treatment immediately post surgery in a preclinical model of anterior cruciate injury inhibited or abolished subsequent development of an osteoarthritis-like/joint damage phenotype in the animals (Barton et al., 2018; Heard et al., 2019; Heard et al., 2022). Similarly, in rat Achilles tendon healing dexamethasone treatment at 7–11 days post-rupture lead to improved material properties of the healing tendon (Dietrich-Zagonel et al., 2018; Dietrich-Zagonel et al., 2022). Interestingly, dexamethasone applied to human tendon cells alter the expression of neuro-inflammatory mediators, i.e., substance P, through a glucocorticoid receptor-dependent pathway (Mousavizadeh et al., 2023). Earlier it has been demonstrated that the peripheral nervous system including pro- and anti-inflammatory neuronal mediators exert essential regulatory functions on tendon healing (Ackermann et al., 2016). Thus, induction of an inflammatory response that is not regulated in a tightly controlled manner, can lead to adverse consequences, likely in the context of injury healing or injury to a soft tissue. While GC treatment may influence the local environment following a connective tissue injury, whether it would be useful in all injury environments remains to be confirmed. Their use may depend on timing, dose and the type of GC employed (Dietrich-Zagonel et al., 2018; Dietrich-Zagonel et al., 2022).

Based on the discussion above, the response of humans to rupture of a tendon such as the Achilles tendon leads to heterogeneity in outcomes, ranging from poor to good. Some of this heterogeneity may reside in the genetic make-up of the patient, but also the environment of the wound site, and potentially whether the rupture is initially repaired surgically or not. Thus, clinical trials focused on assessing the value and impact of an intervention and generated using unselected patient populations would contain both those destined for a good outcome as well as those destined for a poor outcome. This scenario would likely complicate the interpretation of results and any statistical evaluations as an intervention could improve those destined for a poor outcome while not improving those destined for a good outcome. Therefore, what is needed is tools to improve personalized treatment options, and biomarkers that identify subsets of patients could lead to more focused interventions and more directed understanding of the variables contributing to good *versus* poor outcomes. This review is thus focused on that premise.

Furthermore, as a rupture is an acute event, leading to tissue damage and induction of inflammation, the healing process and related events will likely be different from those associated with chronic conditions such as tendinosis and tendinitis. In addition, different tendons exist and function in different biomechanical environments and thus, their biology may also be location-specific and thus some molecular aspects of healing may be unique. Therefore, this review will focus on the ruptured Achilles tendon, but the approaches to identify biomarkers associated with outcomes should be applicable to injuries to other tendons in the future. While the healing of the AT is the major theme of this review, this tendon is used as an example, and the applicability of the approaches used to other tendons is discussed as to whether the findings regarding the healing of mid-substance AT ruptures can be extrapolated to injuries to the AT in other locations and whether the findings can be extended to other tendon injuries is important for the field of repair and regeneration of tendon injuries.

## The wound healing environment after an acute tendon injury

**FIGURE 1:**
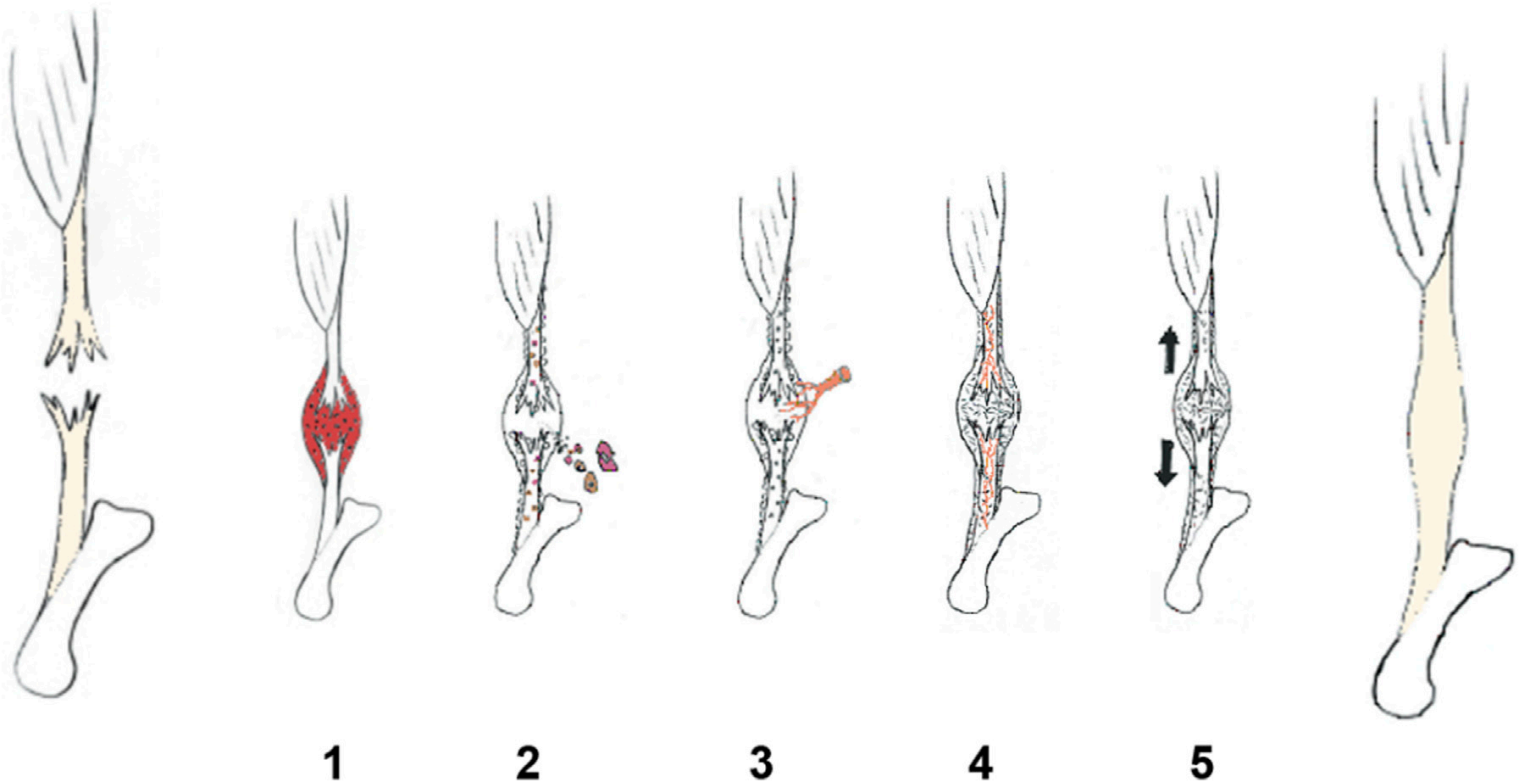
Tendon repair overview (Ackermann and Hart, 2016). (1) Induction (Kwan et al., 2023), (2) production (Svedman et al., 2018), (3) orchestration (Kuroiwa and Amadio, 2023), (4) conduction, and (Wiig et al., 2014) (5) modification of the healing process (Reproduced with permission from Ackermann [Ackermann PW. Healing and repair mechanisms. London: DJO Publications; 2014].

If one suffers a transection or complete rupture of a tendon such as the AT or the flexor tendons of the hand, this injury often requires surgical repair followed by a period of immobilization [discussed in Ackermann et al. (2023); Hart et al. (2023)]. Furthermore, the faster the patients can receive the surgery, the better the outcomes (Svedman et al., 2018). However, in other locals, the leg is merely immobilized for a period of time followed by physiotherapy, and thus in this scenario, the injured tissue is left to its own devices in an immobilized state. In either circumstance, there is initiation of a healing response with a multitude of phases including the inflammatory phase, proliferative phase, matrix deposition phase, and then a prolonged matrix remodeling phase. Alternatively, these phases of healing are labelled as the induction, production, orchestration, and conduction phases of healing (Figure 1).

Even with a surgical intervention to join the torn ends of the AT together, patients can experience a good to excellent outcome at 1-year post-injury, or a poor outcome based on validated criteria [discussed in Chen et al. (2022); Chen et al. (2023); Hart et al. (2023)]. Recently, Chen et al. (2022) and Wu et al. (2023) reported that using proteomic approaches and shards of tissue from the torn ends of ruptured AT taken at the time of surgery led to the identification of biomarkers of good *versus* poor outcomes at 1-year post-injury. Thus, within days of injury, the local environment can predict whether a patient will have a good *versus* poor outcome at 1-year! How such biomarkers translate to good outcomes is currently not well described, but recent reports by Chen et al. (2023a); Chen et al. (2023) indicate that a biomarker of good outcomes, eukaryotic elongation factor-2 (eEF2) can directly affect a number of cell processes and protein expression levels. Whether all of the biomarkers identified directly influence healing outcome or are surrogate markers of outcome remains to be determined. However, the above-described findings indicate that the local environment early after injury to the AT can predict outcomes at 1-year and could help identify patients that may be in need of targeted interventions to improve outcomes. Such targeted interventions may need to be multifaceted, with one facet to enhance the local environment and another to exert a positive influence on the healing process. The reason for indicating a potential need for such an approach is that in the poor outcome patients, one does not really know of the outcome is poor due to a lack of some influence or due to the presence of an inhibitor of a good outcome.

It should be noted that connective tissues such as tendons, and nearly all other tissues of the musculoskeletal system, require mechanical loading to maintain their integrity and subscribe to the “use it or lose it” paradigm [discussed in Hart and Zernicke (2020); Hart (2021); Hart et al. (2022)]. Therefore, if one immobilizes a limb, the muscles and other connective tissues are removed from loading and undergo atrophy. Interestingly, it has been shown with menisci that removal from the knee of the animal leads to the rapid (4 h) induction of a “cassette” of genes that could contribute to catabolism of the tissue, including MMP-1, MMP-3, iNOS, COX-2 and IL-1beta and IL-6, but not MMP-13, collagens, biglycan or TIMP-4 (Natsu-ume et al., 2005). The induction of the expression of this subset of genes could be prevented by *in vitro* administration of intermittent cyclic hydrostatic compression (1 min every 15 min at 1 MPa). Thus, there is a set of genes that are repressed by loading. Whether a similar or different set of genes are also affected by a loss of loading in tendons remains to be determined, however, it is a likely scenario based on responses of individuals with immobilized limbs, prolonged bedrest, or astronauts [discussed in Hart et al. (2022)].

The above discussion is relevant to tendon repair as after surgery, the affected limb is usually immobilized for various periods of time and thus, subjected to conditions that foster atrophy of muscle bone and the surgically repaired AT. This may also affect the vascular system as such patients may incur a deep vein thrombosis at a high rate [∼50%; discussed in Saarensilta et al. (2023b)]. However, this immobilization is occurring after surgery and based on the proteomic studies (Chen et al., 2022; Chen et al., 2023; Wu et al., 2023), biomarkers of good outcomes at 1-year were already evident prior to surgery. It should be noted that the torn AT was already unloaded after the rupture for 2–7 days before surgery, so the torn ends were in fact not only subjected to inflammation-associated with the injury, but also loss of biomechanical loading for several days prior to surgery. And then even after surgery, the limb was immobilized for a period of time. While it is recognized that immobilization is not good for connective tissue health, and the length of the immobilization period should be kept to a minimum so as not to downregulate tendon repair genes (Bring et al., 2009; Bring et al., 2010), with gradual return to minimal loading initially and then increasing as the healing tissue regains strength. Thus, loading is recognized as a positive influence on the healing progression.

While there is still much to understand about what contributes to how the good outcomes are manifested in the early stages after injury, there are at least two processes that are evident, inflammation and loss of loading and its consequences. Early acute inflammation may be very critical to the initial phases of healing and thus a positive (although often viewed as a negative), while the possible catabolic influence of unloading the tissue could be a negative influence on outcomes. Therefore, a good outcome may require some innate ability in the local environment to balance those contributions and regulate their influence to contribute to a good outcome at 1 year. This environment may also influence how effective cellular, biochemical, and drug interventions are to enhance outcomes. One might also expect that the incidence of a good outcome would be associated with early surgery after AT rupture [Svedman et al., 2018), and in those jurisdictions that do not use surgery and only immobilization, there would be fewer good outcomes and more adequate or poor outcomes that may need specific interventions to enhance the quality of the healing process. (Svedman et al., 2018) reported that surgery for an AT rupture within 48 h post-injury led to more good outcomes at 1 year for patients than did those patients receiving surgery >72 h post-injury, although some patients receiving surgery >72 h post-injury still had a good outcome. Therefore, there is some heterogeneity in the response pattern.

Based on the above discussion, there are several options for how the local environment early after a tendon injury may influence long term outcomes at 1-year post-injury. These include: 1) extended time for induction of catabolic atrophy genes before surgery (negative); 2) extended time for development of an inflammatory response with negative elements before surgery; and 3) extended time for an inflammatory response to impact a non-loaded atrophy-induced torn tissue before surgery. It should be noted that the surgical procedure itself is actually a second inflammatory stimulus and thus, can complicate the local environment. These are not mutually exclusive options, and because of human heterogeneity, genetic, epigenetic, and potentially sex-related differences could also influence how the above options evolve in the injured tissue environment and are implemented. However, future studies may have to develop interventions to optimize the local environment to enhance the success of other modalities to improve healing outcomes (Hart and Nakamura, 2022), such as those discussed in later sections of this review.

## Surgical versus non-surgical treatment of at ruptures: Outcomes

The options for treatment after an AT rupture are varied, ranging from immediate surgery to merely casting the affected lower limb in an immobilized state for a period of time, with some variations in between. Immediate surgery will put some tension on the sutured tissue while casting alone will provide a prolonged period of immobilization where the healing process will progress in an initial environment that has no load. Thus, in the latter scenario one may expect that the outcomes at 1-year and beyond would be inferior for such patients compared to those that received immediate surgical repair. However, that is apparently not the case based on the report of Keating and Will (Keating and Will, 2011), but a majority of surgeons prefer surgical treatment for young, active patients (Parisien et al., 2021). In addition, some reports indicate there is a lower rate of re-rupture with surgical repair (Lynch, 2004), potentially indicating that surgical repair leads to better quality repair tissue and/or there is less scar-like repair tissue when the torn ends are sutured. Recently, a high impact multicenter, randomized, controlled trial by Myhrvold et al. confirmed a lower rate of re-rupture with surgical AT repair, although the patient-reported outcome between surgically and non-surgically treated patients showed no differences (Myhrvold et al., 2022). Therefore, patient selection and expectations of participating in an active lifestyle may influence the choice of treatment. In conservative treatment protocols, early mobilization is likely recommended compared to prolonged immobilization via casting (Kangas et al., 2003; Van der Eng et al., 2013), but issues around re-rupture rate and other complications still remain to be resolved in detail. Given the heterogeneity in patient outcomes even within the surgical treatment cohorts [(Svedman et al., 2018); discussed in Hart et al. (2023)], it is also likely, but not proven, that heterogeneity may also exist within the conservative treatment population as well. Thus, comparing two heterogeneous populations within both the surgical and non-surgical groups may lead to an obscuring of differences in outcomes. It may also depend on when the long-term assessments are performed (i.e., 1, 2, 10 years) as a good *versus* poor outcome at 1-year may be overcome by 2 or 10 years dependent on activity level and other parameters. The potential that good outcomes *versus* poor at 1 year is actually based on the rate of healing to yield a good outcome and this may be obscured at 2 or 10 years as those with an initial poor outcome progress to what is now a good outcome. Some of these issues may also depend on the age and sex of the cohorts assessed as a younger population may use the repaired/healed AT differently than an older patient population.

## Attempts to improve outcomes after a tendon injury: Focusing on the at

The average healing of a ruptured tendon, including the AT, is quite variable, leading to the conclusion that they do not heal well. In part, this is due to the fact that the tendon is healing in an environment that is very different from that in which it developed during fetal life [discussed in (He et al., 2022)], and thus expecting complete regeneration may be an unreasonable expectation. However, tendons do contain cells with stem cell-like properties [(Lu et al., 2023)], but their role in healing is not well characterized. In the adult stage of life, how the tendon heals may in part be due to whether it is surgically repaired or just immobilized, but as discussed above, some patients heal with a good outcome while others heal with a poorer outcome at 1-year post-injury. Prior to the reports of good *versus* poor outcomes following natural healing of the AT, and continuing to today, many studies have attempted to improve healing using a variety of interventions without attempting to segregate naturally occurring good and poor healers. While not all of the interventions have assessed efficacy for AT healing, the interventions utilized include growth factors [reviewed in (El-Sherif et al., 2023; Lin et al., 2023; Miescher et al., 2023; Rieber et al., 2023; Wang and Li, 2023)], acupuncture (Stewman, 2023), platelet-rich plasma (PRP) and variations (Markazi et al., 2022; Everts et al., 2023), other cell therapies including mesenchymal stem cells (MSC) (Chamberlain et al., 2017; Alt et al., 2021; Zhang et al., 2021; Jiang L. et al., 2023; Yuan et al., 2023; Zulkifli et al., 2023), and extracellular vesicles (EV) derived from MSC and related stem cells (Lu et al., 2021; Lyu et al., 2022; Wang and Li, 2023; Xue et al., 2023; Zou et al., 2023) and other cellular preparations (Aydin et al., 2023). Use of glucocorticoid injections to ostensibly control inflammation to enhance tendon healing or improve the local injury environment was variable and dependent on a variety of factors (Dietrich-Zagonel et al., 2018; Dietrich-Zagonel et al., 2022), and was often detrimental to healing (Dean et al., 2014). Some of these approaches have been used for treatment of tendinopathies other than ruptures, and in such cases, the outcomes are more related to pain rather than tissue regeneration.

While some of the approaches to improve tendon healing are still experimental in preclinical models, attempts to enhance tendon healing with some interventions such as PRP have been reported to not enhance tendon healing in a significant manner (Keene et al., 2022). However, PRP is used in an autologous manner so the failure to enhance healing could be due to limitations related to the source of the PRP or the local injury environment they were injected into, the age of the donor, the timing of the injection or the volume. As the preparation of PRP can also vary [discussed in (Kydd and Hart, 2020; Godoi et al., 2022; Bagheri et al., 2023; Everts et al., 2023; Giannotti et al., 2023)], this may also influence outcomes.

While the interventions identified above are quite diverse in their chemical, biochemical and cellular basis, their impact on improving clinical outcomes is variable, in part due to the heterogeneity of the patients and the quality of the local post-injury environment (discussed in the last paragraph of the Introduction section), as well as the fact that nearly all patients receiving cellular interventions (i.e., PRP, stem cells, and other cellular preparations) prefer to receive autologous materials which may not be optimal to impact outcomes [discussed in Kydd and Hart (2020); Hart and Nakamura (2022)]. Going forward, using tools such as biomarkers of good *versus* poor clinical outcomes could enhance the use of some of those interventions identified above to improve the outcomes of selected patient subsets (Hart et al., 2023).

Of the cellular interventions discussed above, likely the approach that may offer the best opportunity to improve healing is the use of extracellular vesicles (EVs) that exhibit low immunogenicity (Sarcinella et al., 2023) and thus can be optimized for allogeneic use. EV contain a variety of molecules including miRNAs (Ragni et al., 2020; Ragni et al., 2021; F-Palama et al., 2023) which are reported to influence tendon healing (Liu et al., 2021). The effectiveness of EV can also be influenced by the culture conditions (Hanai et al., 2023; Phelps et al., 2023) and thus, potentially targeted for specific applications.

## The way forward and the next steps

The finding of biomarkers prognostic for good *versus* poor long-term tendon healing outcomes can change the approaches to clinical trials, as well as clinical research. Identifying such biomarkers within days of injury also has implications for how one approaches the evaluation of interventions with proteins, drugs or cellular therapies. The ability to identify biomarkers which relate to outcomes at the different phases of healing should enhance the evaluation of interventions to improve outcomes (summarized in Table 1). As outlined in Table 1, the process of healing is complex so having biomarkers at different stages of the process to assist in such evaluations may be critical. Some approaches to address current gaps in our knowledge and understanding of the process are outlined below.

**TABLE 1:** Healing phases, novel biomarkers of tendon repair and established approaches to enhance tendon repair.

Healing phase	Specification of healing phase	Novel biomarkers. Association with healing outcome	Various approaches to enhance tendon repair
1. Induction	Inflammation. Blood-derived cells, which subsequently releases growth factors	*ITIH4* Higher ITIH4 levels are positively associated with better clinical outcomes after ATR.	*Platelet rich plasma* Derived from centrifugation of whole blood. Such platelet preparations contain many growth factors and anabolic molecules
2. Production	Proliferation.Tissue-derived cells are attracted and transformed into myofibroblasts at the healing site. The myofibroblasts subsequently activate production of tendon callus	*eEF2* Higher eEF2 levels are positively associated during both inflammatory and proliferative healing with improved clinical outcomes after ATR.	*Stem cells,* E.g., Mesenchymal stem cells (MSC) (Hart et al., 2022), bone marrow stem cells (BMSC) (Natsu-ume et al., 2005), and genetically modified cells that synthesize and deliver the desired growth factor in a temporally and spatially orchestrated manner. *Growth factors* IGF TGF-B BMP VEGF
3. Orchestration	Proliferation. New pathways for delivery of healing substances are built with neuro-vascular ingrowth into a tendon-matrix normally devoid of nerves and vessels	*CFD* Lower CFD levels are associated with improved patient outcomes after ATR.	*NGF* and *neuropeptides* are released, which guide neurovascular ingrowth. Subsequently to healing factors that regulate nerve and blood vessel retraction are released. *Early mobilization* accelerates the nerve plasticity, i.e., nerve regeneration, expression of neuromediators and their receptors, and nerve retraction (Lu et al., 2023; Miescher et al., 2023)
4. Conduction	Proliferation. A prerequisite for healing to commence and to initiate the development of a functioning tissue matrix into which cells, vessels, and nerves can grow in and where production of new granulation tissue can occur	*FGF-2* Higher FGF-2 gene expression is positively associated with better patient outcomes after ATR.	Tendon tissue, flap techniques, or *tendon grafts* are used. *Scaffolding techniques*–either biogenic or synthetic (e.g., bioresorbable polymers) scaffolds
5. Modification	Remodeling. Transition from Col III to Col I is essential for scar maturation. Increasing mechanical loading activates myofibroblasts and fibroblasts to increase the production of relevant matrix molecules leading to structural reorganization to enhance the capacity of the tissue to withstand high mechanical load	*Pyruvate.* Higher pyruvate levels are associated with better patient outcomes after ATR.	*Pyruvate* is involved in tendon repair associated with the transition from Col III to Col I in the scar tissue during remodeling. *Increasing mechanical loading* activates myofibroblasts and fibroblasts to increase the production of collagen type I to increase the callus size and enhance the capacity to withstand high mechanical load

### Clinical research

A. Identification of biomarkers via proteomics is really a first step. One next has to determine how the biomarkers are affecting outcomes, or whether they are just surrogates for outcomes. One option of what is needed has been reported by Chen et al. (2023a); Chen et al. (2023), where how one of the biomarkers identified as being related to good healing outcomes (eEF-2) affects cellular processes was investigated. In addition, morphological and immunolocalization studies (I.e., proteins and cells) are needed to assess where the biomarker may be exerting an effect on healing. In addition, one may want to include the use of approaches such as Shear Wave Propagation (Blank et al., 2022) to assess the progression of the healing process from early to later (i.e., 1, 2, 5 years post-surgery) to assess the remodeling stage of healing and whether it is accelerated in some patients compared to those with poor outcomes.
B. The biomarkers identified as prognostic for good vs. poor outcome at 1-year after AT rupture was focused on patients with mid-substance injuries. Are the same biomarkers associated with outcomes after an injury to the myotendinous junction or the insertion into bone? The environments and tissues involved in such injuries are very different from those involved in mid-substance injuries.
C. Do injuries to other tendons (i.e., flexor tendons, supraspinatus, patellar) that require surgical interventions yield similar biomarkers to those identified for AT ruptures, or are they different. Likely both the environments and the tissues/cells are different so one may need to perform studies similar to those reported for the ruptured AT with patients suffering from injuries to other tendons.
D. Are the biomarkers identified as being prognostic of good vs. poor outcomes after AT rupture characteristic of general healing processes (i.e., ligament, skin, etc.) or unique to the ruptured AT? That is, are some people “good healers” and other “poor healers” irrespective of the healing site? This might imply that there is a strong genetic component to the process.

### Clinical trials

A. Clinical trials designed to assess the impact of specific interventions on outcomes could target those destined to achieve good outcomes vs. a poor outcome even after the fact so as to determine whether the two populations would be influenced differently. Thus, converting those destined for a poor outcome to a good outcome could be one set of outcomes for a specific intervention and whether an intervention could further improve those destined for a good outcome naturally would be a second goal of the trial. The results from the proteomics studies could be done independently from the intervention so as not to impact of the timing of the intervention and could also be done in a blinded manner.
B. As discussed previously, healing outcomes likely reflect the effectiveness of repair processes and do not lead to regeneration. Therefore, if regeneration is the goal of the research, then it may be more appropriate to investigate the potential for regeneration with those already destined for a good outcome *versus* those destined for a poor outcome as in the latter, one may have to also overcome deficiencies that the intervention is not capable of addressing.
C. Clinical trials should also be undertaken to determine whether the same of different biomarkers are identified with good vs. poor outcomes depending on the location of the injury in a tendon such as the Achilles tendon. Injuries to this tendon can occur at the bone-tendon interface or at the myotendinous junction, as well as the mid-substance which the current biomarkers have been associated with thus far. As reports indicate that healing outcomes are better the more proximal the injury to the Achilles tendon (Qureshi et al., 2023), this will be important to establish whether separate biomarkers are associated with outcomes following injury at these transition points in the tendon *versus* within the tendon mid-substance.

Thus, the advent of biomarkers of outcomes after a tendon rupture may impact both clinical research and clinical trials in new ways and with enhanced potential to yield benefits to both understanding healing processes and patients in their post-injury life.

## Conclusion

Wound healing after an extensive injury is complex, involving many steps in the process. Healing in tissues designed to function in high load environments, are particularly complex. Therefore, the finding of a number of biomarkers prognostic of good *versus* poor outcomes has the potential to change the way some clinical research and clinical trials are conducted. However, even when biomarkers prognostic for healing outcomes are identified, it is critical to determine how the biomarkers impact healing, how the local environment affects outcomes, and whether one can utilize the information to further enhance the healing potential of specific patient subpopulations. Thus, do biomarkers reflect a critical stage of healing and therefore, an important initial step (s) in this complex process, or are they surrogates for some other process that can be deduced by network analysis of the proteomic data? The answers to such questions are critical to moving the field forward and will require significant new research efforts before they can translate to patient populations. However, these are achievable goals with the right investment and commitment in the not so distant future.

## References

[B1] Ackermann P. W. Ahmed A. S. Hart D. A. (2023). “Medical considerations in tendinopathy,” in Tendon regeneration: understanding tissue physiology and development to engineer functional substitutes. Editors Gomes M. E. Reis R. Rodrigues M. T. Goncalves A. Zeugolis D. Docheva D. 2nd Edition (Elsevier). In Press.

[B2] Ackermann P. W. Hart D. A. (2016). General overview and summary of concepts regarding tendon disease topics addressed related to metabolic disorders. Adv. Exp. Med. Biol. 930, 293–298. 10.1007/978-3-319-33943-6_28 27535271

[B3] Ackermann P. W. Salo P. Hart D. A. (2016). Tendon innervation. Adv. Exp. Med. Biol. 920, 35–51. 10.1007/978-3-319-33943-6_4 27535247

[B4] Addevico F. Svedman S. Edman G. Ackerman P. W. (2019). Pyruvate and lactate as local prognostic biomarkers of patient outcome after Achilles tendon rupture. Scand. J. Med. Sci. Sports. 29, 1529–1536. 10.1111/sms.13469 31102560

[B5] Alt E. Rothoerl R. Hoppert M. Frank H.-G. Wuerfel T. Alt C. (2021). First immunohistochemical evidence of human tendon repair following stem cell injection: a case report and review of literature. World J. Stem cells. 13, 944–970. 10.4252/wjsc.v13.i7.944 34367486 PMC8316863

[B6] Aydin E. Y. Asik M. Aydin H. M. Cay N. Gumuskaya B. Caglayan A. (2023). The co-use of stromal vascular fraction and bone marrow concentrate for tendon healing. Curr. Stem. Cell Res. 18, 1150–1159. 10.2174/1574888X18666230221141743 36803277

[B7] Bagheri K. Krez A. Anastasio A. T. Adams S. B. (2023). The use of platelet-rich plasma in pathologies of the foot and ankle: a comprehensive review of the recent literature. Foot Ankle Surg. 26 (23), 551–559. 10.1016/j.fas.2023.07.010 37516651

[B8] Barton K. I. Heard B. J. Sevick J. L. Martin C. R. Shekarforoush S. M. M. Chung M. (2018). Posttraumatic osteoarthritis development and progression in an ovine model of partial anterior cruciate ligament transection and effect of repeated intra-articular methylprednisolone acetate injections on early disease. Am. J. Sports Med. 46, 1596–1605. 10.1177/0363546518765098 29668309

[B9] Blank J. Blomquist M. Arant L. Cone S. Roth J. (2022). Characterizing musculoskeletal tissue mechanics based on shear Wave propagation: a systematic review of current methods and reported measurements. Ann. Biomed. Eng. 50, 751–768. 10.1007/s10439-022-02935-y 35359250 PMC9631468

[B10] Bring D. Reno C. Renstrom P. Salo P. Hart D. Ackermann P. (2010). Prolonged immobilization compromises up-regulation of repair genes after tendon rupture in a rat model. Scand. J. Med. Sci. Sports. 20, 411–417. 10.1111/j.1600-0838.2009.00954.x 19602192

[B11] Bring D. K.-I. Reno C. Renstrom P. Salo P. Hart D. A. Ackermann P. W. (2009). Joint immobilization reduces the expression of sensory neuropeptide receptors and impairs healing after tendon rupture in a rat model. J. Orthop. Res. 27, 274–280. 10.1002/jor.20657 18655130

[B12] Chamberlain C. S. Saether E. E. Aktas E. Vanderby R. (2017). Mesenchymal stem cell therapy on tendon/ligament healing. J. Cytokine Bio. 2, 112. 10.4172/2576-3881.1000112 28670649 PMC5493432

[B13] Chen J. Svensson J. Sundberg C.-J. Ahmed A. S. Ackermann P. W. (2021). FGF gene expression in injured tendons as a prognostic biomarker of 1-year patient outcome after Achilles tendon repair. J. Exp. Orthop. 8, 20. 10.1186/s40634-021-00335-0 33694106 PMC7947072

[B14] Chen J. Wang J. Hart D. A. Ahmed A. S. Ackermann P. W. (2022). Complement factor D as a predictor of Achilles tendon healing and long-term patient outcomes. FASEB J. 36, e22365. 10.1096/Fj.202200200RR 35596679

[B15] Chen J. Wang J. Hart D. A. Zhou Z. Ackermann P. W. Ahmed A. S. (2023a). Complement factor D regulates collagen type I expression and fibroblast migration to enhance human tendon repair and healing outcomes. Front. Immunol. 14, 1225957. 10.3389/fimmu.2023.1225957 37744351 PMC10512081

[B16] Chen J. Wang J. Wu X. Simon N. Svensson C. I. Yuan J. (2023). eEF2 improves dense connective tissue repair and healing outcome by regulating cellular death, autophagy, apoptosis, proliferation and migration. Cell Mol. Life Sci. 80, 128. 10.1007/s00018-023-04776-x 37084140 PMC10121543

[B17] Dean B. J. F. Franklin S. L. Murphy R. J. Javaid M. K. Carr A. J. (2014). Glucocorticoids induce specific ion-channel-mediated toxicity in human rotator cuff tendon: a mechanism underpinning the ultimately deleterious effect of steroid injection in tendinopathy? Br. J. Sports Med. 48, 1620–1626. 10.1136/bjsports-2013-093178 24677026

[B18] Dietrich-Zagonel F. Aspenberg P. Eliasson P. (2022). Dexamethasone enhances Achilles tendon healing in an animal injury model, and the effects are dependent on dose, administration time, and mechanical loading stimulation. Am. J. Sports Med. 50, 1306–1316. 10.1177/03635465221077101 35234541 PMC9014685

[B19] Dietrich-Zagonel F. Mannerman M. Tatting L. Dietrich F. Ljunggren M. K. Blomgran P. (2018). Stimulation of tendon healing with delayed dexamethasone treatment is modified by the microbiome. Am. J. Sports Med. 46, 3281–3287. 10.1177/0363546518799442 30265844

[B20] Edsfeldt S. Holm B. Mahlapuu M. Reno C. Hart D. A. Wiig M. (2017). PXL01 in sodium hyaluronate results in increased PRG4 expression: a potential mechanism for anti-adhesion. Ups. J. Med. Sci. 122, 28–34. 10.1080/03009734.2016.1230157 27658527 PMC5361429

[B21] El-Sherif S. M. Abdel-Hamid M. M. Noureldin J. M. A. M. Fahmy H. M. Abdel-Naby H. M. A. (2023). Effectiveness of lyophilized growth factors injection for subacromial impingement syndrome: a prospective randomized double-blind placebo-controlled study. J. Orthop. Surg. Res. 18, 78. 10.1186/s13018-023-03548-4 36721157 PMC9887845

[B22] Everts P. A. Lana J. F. Onishi K. Buford D. Peng J. Mahmood A. (2023). Angiogenesis and tissue repair depend on platelet dosing and bioformulation strategies following orthobiological platelet-rich plasma procedures: a narrative review. Biomedicines 11, 1922. 10.3390/biomedicines11071922 37509560 PMC10377284

[B23] F-Palama M. E. Coco S. Shaw G. M. Reverberi D. Ghelardoni M. Ostano P. (2023). Xeno-free cultured mesenchymal stromal cells release extracellular vesicles with a “therapeutic” miRNA cargo ameliorating cartilage inflammation *in vitro* . Theranostics 13, 1470–1489. 10.7150/thno.77597 37056573 PMC10086204

[B24] Geremia J. M. Bobberrt M. F. Nova M. C. Ott R. D. de Aguiar Lemos F. de Oliverira Lupion R. (2015). The structural and mechanical properties of the Achilles tendon 2 years after surgical repair. Clin. Biomech. (Bristol, Avon) 30, 485–492. 10.1016/jclinbiomech.2015.03.005 25828432

[B25] Giannotti L. Stanca B. D. C. Spedicato F. Nitti P. Damiano F. Demitri C. (2023). Progress in regenerative medicine: exploring autologous platelet concentrates and their clinical applications. Genes (Basel). 14, 1669. 10.3390/genes14091669 37761809 PMC10530962

[B26] Godoi T. T. F. Rodrigues B. L. Huber S. C. Santana M. H. A. da Fonseca L. F. Santos G. S. (2022). Platelet-rich plasma gel matrix (PRP-GM): description of a new technique. Bioeng. (Basel). 9, 817. 10.3390/bioengineering9120817 PMC9774306 36551023

[B27] Hanai H. Hart D. A. Jacob G. Shimomura K. Ando W. Yoshioka Y. (2023). Small extracellular vesicles derived from human adipose-derived mesenchymal stromal cells cultured in a new chemically-defined contaminate-free media exhibit enhanced biological and therapeutic effects on human chondrocytes *in vitro* and in a mouse osteoarthritis model. J. Extracell. Vesicles. 12, e12337. 10.1002/jev2.12337 37367299 PMC10295161

[B28] Hart D. A. (2021). Learning from human responses to deconditioning environments: improved understanding of the “use it or lose it” principle. Front. Sports Act. Living. 3, 685845. 10.3389/fspor.2021.685845 34927066 PMC8677937

[B29] Hart D. A. Ahmed A. S. Ackermann P. (2023). Optimizing repair of tendon ruptures and chronic tendinopathies: integrating the use of biomarkers with biological interventions to improve patient outcomes and clinical trial design. Front. Sports Act. Living. 4, 1081129. 10.3389/fspor.2022.1081129 36685063 PMC9853460

[B30] Hart D. A. Nakamura N. (2022). Creating an optimal *in vivo* environment to enhance outcomes using cell therapy to repair/regenerate injured tissues of the musculoskeletal system. Biomedicines 10, 1570. 10.3390/biomedicines10071570 35884875 PMC9313221

[B31] Hart D. A. Zernicke R. F. (2020). Optimal human functioning requires exercise across the lifespan: mobility in a 1g environment is intrinsic to the integrity of multiple biological systems. Front. Physiol. 11, 156. 10.3389/fphys.2020.00156 32174843 PMC7056746

[B32] Hart D. A. Zernicke R. F. Shrive N. G. (2022). *Homo sapiens* may incorporate daily acute cycles of “conditioning-deconditioning” to maintain musculoskeletal integrity: need to integrate with biological clocks and circadian rhythm mediators. Int. J. Mol. Sci. 23, 9949. 10.3390/ijms23179949 36077345 PMC9456265

[B33] He P. Ruan D. Huang Z. Wang C. Xu Y. Cai H. (2022). Comparison of tendon development versus tendon healing and regeneration. Front. Cell Dev. Biol. 10, 821667. 10.3389/fcell.2022.821667 35141224 PMC8819183

[B34] Heard B. J. Barton K. I. Abubacker S. Chung M. Martin C. R. Schmidt T. A. (2022). Synovial and cartilage responsiveness to peri-operative hyaluronic acid +/_ dexamethasone administration following limited injury to the rabbit stifle joint. J. Orthop. Res. 40, 8380845. 10.1002/jor.25108 34061360

[B35] Heard B. J. Barton K. I. Agbojo O. M. Chung M. Sevick J. L. Bader T. J. (2019). Molecular response of rabbit menisci to surgically induced hemarthrosis and a single intra‐articular dexamethasone treatment. J. Orthop. Res. 37, 2043–2052. 10.1002/jor.24346 31095777

[B36] Jiang K. Li Y. Xiang C. Xiong Y. Jia J. (2023a). Rebalancing SMAD7/SMAD3 signaling reduces adhesion formation during flexor tendon healing. J. Microbiol. Biotechnol. 33, 339–347. 10.4014/jmb.2209.09033 36859314 PMC10084761

[B37] Jiang L. Lu J. Chen Y. Lyu K. Long L. Wang X. (2023b). Mesenchymal stem cells: an efficient cell therapy for tendon repair (review). Int. J. Mol. Med. 52, 70. 10.3892/ijmm.2023.5273 37387410 PMC10373123

[B38] Kangas J. Pajala A. Siira P. Hamalainen M. Leppilahti J. (2003). Early functional treatment versus early immobilization in tension of the musculotendinous unit after Achilles rupture repair: a prospective, randomized, clinical study. J. Trauma. 54, 1171–1180. 10.1097/01.ta.0000047945.20863.a2 12813340

[B39] Keating J. F. Will E. M. (2011). Operative versus non-operative treatment of acute rupture of tendo Achillis: a prospective randomized evaluation of functional outcome. J. Bone Jt. Surg. Br. 93, 107101078. 10.1302/0301-620X.93B8.25998 21768631

[B40] Keene D. J. Alsousou J. Harrison P. O’Conner H. M. Wagland S. Dutton S. J. (2022). Platelet-rich plasma injection for acute Achilles tendon rupture. Bone Jt. J. 104-B, 1256–1265. 10.1302/0301-620x.104b11.bjj-2022-0653.r1 PMC9621093 36317349

[B41] Kuroiwa T. Amadio P. C. (2023). Flexor tendon adhesion formation: current concepts. Hand Clin. 39, 171–180. 10.1016/j.hcl.2022.08.018 37080649

[B42] Kwan K. Y. C. Ng K. W. K. Rao Y. Zhu C. Qi S. Tuan R. S. (2023). Effect of aging on tendon biology, biomechanics and implications for treatment approaches. Int. J. Mol. Sci. 24, 15183. 10.3390/ijms242015183 37894875 PMC10607611

[B43] Kydd A. S. R. Hart D. A. (2020). Efficacy and safety of platelet-rich plasma injections for osteoarthritis. Curr. Treat. Options Rheum. 6, 87–98. 10.1007/s40674-020-00142-1

[B44] Lantto I. Heikkinen J. Flinkkila T. Ohtonen P. Kangas J. Siira P. (2015). Early functional treatment versus cast immobilization in tension after achilles rupture repair: results of a prospective randomized trial with 10 or more years of follow-up. Am. J. Sports Med. 43, 2302–2309. 10.1177/0363546515591267 26229048

[B45] Lin M. Li W. Ni X. Sui Y. Li H. Chen X. (2023). Growth factors in the treatment of Achilles tendon injury. Front. Bioeng. Biotechnol. 11, 1250533. 10.3389/fbioe.2023.1250533 37781529 PMC10539943

[B46] Liu Q. Zhu Y. Zhu W. Zhang G. Yang Y. P. Zhao C. (2021). The role of microRNAs in tendon injury, repair and related tissue engineering. Biomaterials 277, 121083. 10.1016/j.biomaterials.2021.121083 34488121 PMC9235073

[B47] Lu J. Chen H. Lyu K. Jiang L. Chen Y. Long L. (2023). The functions and mechanisms of tendon stem/progenitor cells in tendon healing. Stem Cells Int. 2023, 1–18. 10.1155/2023/1258024 PMC10509002 37731626

[B48] Lu V. Tennyson M. Zhang J. Khan W. (2021). Mesenchymal stem cell-derived extracellular vesicles in tendon and ligament repair-a systematic review of *in vivo* studies. Cells 10, 2553. 10.3390/cells10102553 34685532 PMC8533909

[B49] Lynch R. M. (2004). Achilles tendon rupture: surgical versus non-surgical treatment. Accid. Emerg. Nurs. 12, 149–158. 10.1016/j.aaen.2003.11.004 15234712

[B50] Lyu K. Liu T. Chen X. Lu J. Jiang L. Liu X. (2022). A “cell-free treatment” for tendon injuries: adipose stem cell-derived exosomes. Eur. J. Med. Res. 27, 75. 10.1186/s40001-022-00707-x 35643543 PMC9148514

[B51] Markazi R. Soltani-Zangbar M. S. Zamani M. Eghbal-Fard S. Motavalli R. Kamrani A. (2022). Platelet lysate and tendon healing: comparative analysis of autologous frozen-thawed PRP and ketorolac tromethamine in the treatment of patients with rotator cuff tendinopathy. Growth factors. 40, 163–174. 10.1080/08977194.2022.2093198 36026559

[B52] Miescher I. Rieber J. Calcagni M. Buschmann J. (2023). *In vitro* and *in vivo* effects of IGF-1 delivery strategies on tendon healing: a review. Int. J. Mol. Sci. 24, 2370. 10.3390/ijms24032370 36768692 PMC9916536

[B53] Mousavizadeh R. Backman L. McCormack R. G. Scott A. (2023). Dexamethasone decreases substance P expression in human tendon cells: an *in vitro* study. Rheumatol. Oxf. 54, 318–323. 10.1093/rheumatology/keu315 PMC4301709 25150176

[B54] Myhrvold S. B. Brower E. F. Andresen T. K. M. Rydevik K. Amundsen M. Grun W. (2022). Nonoperative or surgical treatment of acute Achilles’ tendon rupture. N. Engl. J. Med. 386, 1409–1420. 10.1056/NEJMoa2108447 35417636

[B55] Natsu-ume T. Majima T. Reno C. Shrive N. G. Frank C. B. Hart D. A. (2005). Menisci of the rabbit knee require mechanical loading to maintain homeostasis: cyclic hydrostatic compression *in vitro* prevents derepression of catabolic genes. J. Orthop. Sci. 10, 396–405. 10.1007/s00776-005-0912-x 16075173

[B56] Parisien R. L. Trofa D. P. Gualtien A. P. Dodson C. C. Li X. Levine W. N. (2021). How do sports medicine and foot and ankle specialists treat acute Achilles tendon ruptures? Foot Ankle Spec. 14, 114–119. 10.1177/1938640019901055 31971006

[B57] Phelps J. Hart D. A. Mitha A. P. Duncan N. A. Sen A. (2023). Physiological oxygen conditions enhance the angiogenic properties of extracellular vesicles from human mesenchymal stem cells. Stem Cells Res. Ther. 14, 218. 10.1186/s13287-023-03439-9 PMC10463845 37612731

[B58] Qureshi A. Gulati A. Adukia V. Shah A. Mangwani J. (2023). The influence of the site of rupture and gap distance in acute Achilles tendon rupture treated with functional rehabilitation. Injury 54, 1216–1221. 10.1016/j.injury.2023.02.020 36828734

[B59] Ragni E. Orfei C. P. Silini A. R. Colombini A. Vigano M. Parolini O. (2020). miRNA reference genes in extracellular vesicles released from amniotic membrane-derived mesenchymal stromal cells. Pharmaceutics 12, 347. 10.3390/pharmaceutics12040347 32290510 PMC7238137

[B60] Ragni E. papait A. Orfei C. P. Silini A. R. Colombini A. Vigano M. (2021). Amniotic membrane-mesenchymal stromal cells secreted factors and extracellular vesicle-miRNAs: anti-inflammatory and regenerative features for musculoskeletal tissues. Stem Cells Transl. Med. 10, 1044–1062. 10.1002/sctm.20-0390 33656805 PMC8235131

[B61] Rieber J. Meier-Burgisser G. Miescher I. Weber F. E. Wolint P. Yao Y. (2023). Bioactive and elastic emulsion electrospun DegraPol tubes delivering IGF-1 for tendon rupture repair. Int. J. Mol. Sci. 24, 10272. 10.3390/ijms241210272 37373418 PMC10299220

[B62] Saarensilta A. Aufwerber S. Silbernagel K. G. Ackermann P. W. (2023a). Early tendon morphology as a biomarker of long-term patient outcomes after surgical repair of Achilles tendon rupture: a prospective cohort study. Orthop. J. Sports Med. 11, 23259671231205326. 10.1177/23259671231205326 37941888 PMC10629330

[B63] Saarensilta A. Chen J. Reitzner S. Hart D. A. Ahmed A. Ackermann P. W. (2023b). Novel tissue biomarkers predict deep vein thrombosis and healing outcomes after Achilles tendon rupture. Submitted. 10.1038/s41598-025-91511-0 PMC11873306 40025102

[B64] Sarcinella A. Femmino S. Brizzi M. F. (2023). Extracellular vesicles: emergent and multiple sources in wound healing treatment. Int. J. Med. Sci. 24, 15709. 10.3390/ijms242115709 PMC10650196 37958693

[B65] Stewman C. G. (2023). Ultrasound-guided electroacupuncture treatment for rotator cuff tendinopathy: proposing an effective alternative to nonoperative medical treatments. Med. Acupunct. 35, 257–261. 10.1089/acu.2023.0042 37900871 PMC10606951

[B66] Svedman S. Juthberg R. Edman G. Ackermann P. W. (2018). Reduced time to surgery improves patient-reported outcome after Achilles tendon rupture. Am. J. Sports Med. 46, 2929–2934. 10.1177/0363546518793655 30169112

[B67] Van der Eng D. M. Schepers T. Goslings J. C. Schep N. W. L. (2013). Rerupture rate after early weightbearing in operative versus conservative treatment of Achilles tendon ruptures: a meta-analysis. J. Foot Ankle Surg. 52, 622–628. 10.1053/j.jfas.2013.03.027 23659914

[B68] Wang Y. Li J. (2023). Current progress in growth factors and extracellular vesicles in tendon healing. Int. Wound J. 20, 3871–3883. 10.1111/iwj.14261 37291064 PMC10588330

[B69] Wiig M. Dahlin L. B. Friden J. Hagberg L. Larsen S. E. Wiklund K. (2014). PXL01 in sodium hyaluronate for improvement of hand recovery after flexor tendon repair surgery: randomized controlled trial. PLoS One 9, e110735. 10.1371/journal.pone.0110735 25340801 PMC4207831

[B70] Wu X. Chen J. Sun W. Hart D. A. Ackermann P. W. Ahmed A. S. (2023). Network proteomic analysis identifies inter-alpha-trypsin inhibitor heavy chain 4 during early human Achilles tendon healing as a prognostic biomarker of good long-term outcomes. Front. Immunol. 14, 1191536. 10.3389/fimmu.2023.1191536 37483617 PMC10358850

[B71] Xue Y. Riva N. Zhao L. Shieh J.-S. Chin Y.-T. Gatt A. (2023). Recent advances of exosomes in soft tissue injuries in sports medicine: a critical review on biological and biomaterial applications. J. Control Release 364, 90–108. 10.1016/j.jconrel.2023.10.031 37866405

[B72] Yuan Z. Yu H. Long H. Dai Y. Shi L. Zhao J. (2023). Stem cell applications and tenogenic differentiation strategies for tendon repair. Stem Cells Int. 2023, 1–15. 10.1155/2023/3656498 PMC10033217 36970597

[B73] Zhang Z. Li Y. Zhang T. Shi M. Song X. Yang S. (2021). Hepatocyte growth factor-induced tendon stem cell conditioned medium promotes healing of injured Achilles tendon. Front. Cell Dev. Biol. 9, 654084. 10.3389/fcell.2021.654084 33898452 PMC8059769

[B74] Zou J. Yang W. Cui W. Li C. Ma C. Ji X. (2023). Therapeutic potential and mechanisms of mesenchymalstem cell-derived exosomes as bioactive materials in tendon-bone healing. J. Nanobiotechnology. 21, 14. 10.1186/s12951-023-01778-6 36642728 PMC9841717

[B75] Zulkifli A. Ahmad R. E. Krishnan S. Kong P. Nam H. Y. Kamarul T. (2023). The potential mechanism of hypoxia-induced tenogenic differentiation of mesenchymal stem cell for tendon regeneration. Tissue Cell 82, 102075. 10.1016/j.tice.2023.102075 37004269

